# Virtuous Collective Attention

**DOI:** 10.1007/s11245-024-10040-z

**Published:** 2024-05-03

**Authors:** Isabel Kaeslin

**Affiliations:** University of Fribourg, Fribourg, Switzerland

**Keywords:** Attention, Collective action, Collectives, Virtue, Hermeneutic challenges, Institutionalization, Embodiment, Practical identities

## Abstract

How can a collective pay attention virtuously? Imagine a group of scientists. It matters what topics they pay attention to, that is, which topics they draw to the foreground and take to be relevant, and which they leave in the background. It also matters which aspects of an investigated phenomenon they foreground, and which aspects they leave unnoticed in the background. If we want to understand not only how *individuals* pay attention of this kind virtuously, but also *collectives*, we first need a framework to understand virtuous collective agency. A result of this article will be that virtuous collective action depends on the collective being *institutionalized*. At the same time, we have to think of the constituents of the collective in terms of *practical identities* (as opposed to individuals). This is what enables us to understand how a collective can acquire the stability required for virtue, and how we don’t end up with a summative account of group virtue, respectively. It will be argued that collectives only have the required stability in their actions when their commitments are habitualized in the form of institutionalized procedures. An Aristotelian understanding of virtue distinguishes between commitment, inclination, and action. Only when a subject’s inclination is fully lined up with her commitment, do we arrive at the required stability (of character) for virtuous action. In the case of individuals, to build up an appropriate inclination consists in an inscribing of the commitment into the feelings and body of the subject. If a commitment is fully ‘embodied’ in this sense, it has formed the individual’s inclination accordingly. How can one make sense of this in the case of collective subjects? This article tries to show that for collectives, the embodiment of commitment (the forming of the fitting inclinations) consists in creating policies, procedures, and rules that stabilize the acting according to the commitment, irrespective of the motivation of each individual involved in the collective. Hence, embodiment of commitment, in the case of collectives, is institutionalization. The article then explores what this requirement of institutionalization means for collective attention. The illustration will draw on a distinction between focused and open-minded attention. It will be shown that for either case – focused and open-minded – in order for a collective to pay attention virtuously, it needs to have its commitments institutionalized.

## Introduction

How can a collective pay attention virtuously? Imagine a group of scientists. It matters what topics they pay attention to, that is, which topics they draw to the foreground and take to be relevant, and which they leave in the background. It also matters which aspects of an investigated phenomenon they foreground, and which aspects they leave unnoticed in the background. If we want to understand not only how *individuals* pay attention of this kind virtuously, but also *collectives*, we first need a framework to understand virtuous collective agency.

A result of this article will be that virtuous collective action depends on the collective being *institutionalized.* At the same time, we have to think of the constituents of the collective in terms of *practical identities* (as opposed to individuals). This is what enables us to understand how a collective can acquire the stability required for virtue, and how we don’t end up with a summative account of group virtue, respectively.

It will be argued that collectives only have the required stability in their actions when their commitments are habitualized in the form of institutionalized procedures. An Aristotelian understanding of virtue distinguishes between commitment, inclination, and action. Only when a subject’s inclination is fully lined up with her commitment, do we arrive at the required stability (of character) for virtuous action. In the case of individuals, to build up an appropriate inclination consists in an inscribing of the commitment into the feelings and body of the subject. If a commitment is fully ‘embodied’ in this sense, it has formed the individual’s inclination accordingly. How can one make sense of this in the case of collective subjects?

This article tries to show that for collectives, the embodiment of commitment (the forming of the fitting inclinations) consists in creating policies, procedures, and rules that stabilize the acting according to the commitment, irrespective of the motivation of each individual involved in the collective. Hence, embodiment of commitment, in the case of collectives, is institutionalization.

The article then explores what this requirement of institutionalization means for collective attention. The illustration will draw on a distinction between focused and open-minded attention. It will be shown that for either case—focused and open-minded—in order for a collective to pay attention virtuously, it needs to have its commitments institutionalized.

## Virtuous Collectives: Joint Commitment and Fitting Inclination

We have to get clear first on how we can think of virtuous collective agency more generally, before we can ask about virtuous collective attention as an instance of it. Miranda Fricker (2010) suggests that there can be *group virtue*, just like groups^1^ can have other features in a non-summative way.^2^ Examples of group virtue are: ‘this research group is very *thorough*’, ‘the jury is *fair-minded’*, ‘the union is very *patient’*.^3^ A group can also have a non-summative group-feature that is not virtue-related. An example would be: ‘The Swiss soccer team is *unpredictable.’* This is neither good nor bad, thus not virtue-related. There can, finally, also be non-summative group *vices*. An example would be: ‘The jury is (knowingly) *racist*.’ Hence, group virtue consists in, on the one hand, a feature that is to be found genuinely on the group level, not on the individual level (more on this below), and on the other hand a positively valenced normative feature.

The broadly Aristotelian notion of virtue that Fricker uses^4^ holds roughly that two conditions need to be met for something to amount to group virtue: 1) there must be a joint commitment by the members of the group to achieve the feature (e.g., thoroughness), and 2) there must be a reliable execution of this commitment (Fricker 2010, pp. 241–244).^5^ This seems to be a good starting point. One challenge is that non-summative accounts of collective agency hold that the actions (or dispositions) of a group are not reducible to the sum of the actions (or dispositions) of the members of the group.^6^ By extension this means that group virtue cannot depend on the current individual commitments. In the following, a response to this challenge will be offered. Again, the challenge is that the group must be ‘committed’ to the virtuous end in a different way than based on the current individual members’ commitments, if it is supposed to be a genuine non-summative account of group virtue. The solution offered in the following consists in the idea that the constituents of a group are practical identities, as opposed to individuals. So far, our account is in line with Fricker’s account of group virtue, and only spells out in a bit more detail just how central the role of practical identities is.

The second challenge is how a group achieves ‘reliable execution’ of its commitment. This aspect is not spelled out in Fricker’s own account at all.^7^ This article argues that it is not a simple matter to think about how a group achieves the reliability or stability required for virtue. While we might easily think of general examples where a group of people has stability (e.g., a group of friends who do some things quite reliably), in the cases where virtue is the goal, it is presumably a difficult-to-achieve feature. That is, while it might be easy to think how a group reliably does something for which the individual members have independent motivation, this becomes remarkably more difficult if we cannot base the reliable execution on the individual’s (ongoing) motivation. It might, for example, be difficult for a group to achieve stability in not being prejudiced. In this article, it is argued that for the proper stability in such cases, the group needs to be institutionalized. Hence, this article improves on the Frickerian account of group virtue in two ways: It makes practical identities even more central and spells out in more detail why we need to assume them in order to arrive at a non-summative account of group virtue. And it makes a further contribution by spelling out, for the first time, *how* the required reliability of a group for a virtuous end can be achieved (which is more difficult than one might have assumed).

### Joint Commitment: The Need for Practical Identities

A practical identity is not the same as an individual. An individual might hold some commitments and motivations as part of one of her practical identities, and different commitments and motivations as part of another practical identity. That is, one and the same individual’s practical identities can clash with each other, or rather, the commitments and values that come with each practical identity can clash. At the same time, one and the same practical identity can be taken up by various individuals.

Imagine, for example, that you have adopted a practical identity as a bike rider, and also another one as a regular at your local café. Whenever you ride your bike across the city, you embody your practical identity as a bike rider. You find it important that people observe traffic rules as part of that practical identity. As a regular at your local café, you have different values. For instance, you find it important that you can walk back and forth from the terrace and the main building in a leisurely way. Whenever you’re at your local café, you embody your practical identity as a regular of that café. We can now imagine that these two practical identities of yours can clash. There is a bike route going past the café where you are a regular. Your local café has a terrace for which you have to cross the bike route. You and the other regulars find it important that they can walk back and forth from the terrace in a leisurely way, without having to look out for those fast passing bike riders. But it is the bike riders’ right of way. So, *as* a regular of the café, you think one should not have to observe the right of way of the bike riders. The bike riders should rather have to look out for the people crossing to the terrace, trying to enjoy their time. But *as* a bike rider you think those café customers should get out of the way, observing the traffic rules like everyone else. The values of your two practical identities clash.

What this shows is that the commitments as part of your practical identities are not identical with your commitments as an individual. That is, you can have stable commitments as part of one practical identity, and stable commitments as part of another one, the commitments of the two can disagree, and none of them are identical with your commitments as an individual.^8^

Let me note here that there is some divergence in the literature on how the notion ‘practical identity’ is understood. The above aligns with Miranda Fricker’s use, and shows a marked difference to how the term was introduced by Christine Korsgaard, for example.^9^ Korsgaard understands ‘practical identity’ as “a description under which you value yourself, a description under which you find your life to be worth living and your actions to be worth undertaking” (Korsgaard 1996, p. 101). The starkest difference between Fricker’s and Korsgaard’s conception of practical identity, which is an important aspect for the issues in this article, is that in Korsgaard’s conception one has, ideally, one main practical identity, a unifying conception of oneself. While, as she says, “for the average person there will be a jumble of such conceptions” (ibid.), one aims for some internal consistency in one’s self-constitution, and self-conceptions that contradict each other are cause for concern. In Fricker’s conception, by contrast, even in a virtuous subject there are several practical identities present in one individual. A practical identity is not thought of as the one unifying conception of oneself. Rather, one individual can inhabit several, even such that are in contradiction with each other, without further concern: “(…) the fact that a person has more than one practical identity, so that with one hat on they believe that p, while with another hat on, they do not believe that p, or even believe that non-p” (ibid., p. 246). This is an important aspect of the term ‘practical identity’ as used in this article, in alignment with Fricker’s conception. It is exactly what allows for the distinction between an individual and her practical identities. It is possible for her to practically identify as a bike rider, and also as a regular at her café. Even though these two practical identities can come into conflict with each other, this does not mean that she, as an individual, is in any way disunified or has a problematic character. It just so happens that we can identify with various roles and collectives, and that they aren’t always consistent with each other. This specification of how to understand ‘practical identity’ in this article is important as it is what allows us to say that group virtue is composed of its component-practical-identities, without having to say that group virtue is composed of virtuous individual commitments. That is, this notion of practical identity allows us to see how group virtue can indeed have components (its practical identities), without it being a summative notion of group virtue. This is so because practical identities, in this conception, are not numerically identical with individuals. They are identities that can be adopted and performed by various individuals, and that can persist side by side with other identities that are adopted and performed by the same individual. Group virtue, as we said, consists in one part in a joint commitment (to the virtuous feature). This commitment, however, cannot come about as a sum of the individual members’ commitments, lest we end up with a summative (non-genuine) notion of group agency. We can, however, think of the joint commitment of a group as being constituted by the commitments of the various *practical identities* that constitute the group, instead of as constituted by the commitments of the individuals involved. Fricker conceived of this as one of several ways in which we can arrive at a non-summative account of group virtue. This article spells out in more detail just how central the idea of practical identities is for a non-summative account of group virtue, to the extent that the other ways to achieve group virtue considered by Fricker might end up not amounting to full group virtue at all according to the here presented account.

Let me illustrate by an example. Let’s say a group (“the department”) consists in the practical identities of, for instance, a few professors, a few grad students, and two administrative staff, and each of the practical identities is committed to achieving *thoroughness in research output* (even if their own task within the group has nothing to do with research). In such a case, we can say that together these practical identities constitute a department (a group) that is committed to thoroughness in research. Note that in such a scenario, while the practical identities that constitute the group stay the same over time, the individuals that ‘fill’ and perform these practical identities can (and most likely will) change. Hence, we have a non-summative account of a collective, thus a genuine collective. We can say that the commitment coming from the constituents of the collective is part of what makes it a *virtuous* collective. One requirement for group virtue is thus that the group consists of practical identities (as opposed to individuals) that are committed to virtues.^10^

With this, we have a coherent account of the first requirement for group virtue, that is, an account of how to think of a *joint but non-summative* commitment to virtue of the group’s constituents. We can now turn to the second requirement for group virtue, that is, reliable execution (of the commitment). Both of these requirements are necessary for group virtue, hence having only one of them would not be sufficient.

### Fitting Inclination: The Need for Institutionalization

In Aristotle’s notion of (individual) virtue, there are three ways of failing to be virtuous: being merely self-controlled, being akratic, and being vicious. What the possibility of these non-virtuous forms of character shows us is that there can be a difference between three things: what one is *committed* to, how one is *inclined* to act, and how one *acts* (execution). Akrasia, weakness of will, is the following: One is committed to a virtuous goal (e.g., to only eat healthy food), one’s character is inclined otherwise (e.g., to eat sweets), and one acts otherwise (one eats the sweets). In self-control, one is committed to a virtuous goal, one’s character is inclined otherwise, but one acts according to one’s commitment and against one’s inclination (e.g., eats the healthy food). The difference between self-control and virtue is ‘only’ that in virtue, one’s inclination already accords with the virtuous commitment – that is, one is already inclined to eat the healthy food, and so it does not require self-control (against one’s inclination) to act according to the virtuous commitment. Aristotle is notorious for saying that only *this* amounts to full virtue—if it takes self-control to act virtuously, then you’re not fully virtuous yet. And then finally, in vice, one is already committed to a vicious goal (e.g., to eat sweets), and one acts accordingly.^11^

This distinction into virtue, self-control, akrasia, and vice, based on different constellations of commitment, inclination (habit), and execution (action), is a standard way how Aristotle has been interpreted.^12^ There is a debate on whether Aristotle really wouldn’t have accepted self-control as an excellent state of character, too—that is, whether the kind of character who commits to the good end (because she is capable of seeing its goodness), and has an inclination to act *against* her commitment, but does, through struggle, ultimately act according to her commitment, really isn’t virtuous according to Aristotle. Agnes Callard (2017), for example, argues that Aristotle has been wrongly interpreted as not seeing this as virtuous, and that the standard view (which she calls the “purist” interpretation) wrongly makes it look as if only the one whose inclination always agrees with her commitment is considered an excellent agent. It’s not possible in the scope of this article to defend the standard or purist interpretation against which Callard argues. The standard interpretation will be assumed here, in what I have called “Aristotelian” virtue. This is not insignificant. One of the main results of the article—that only institutionalized groups can be virtuous—hinges on the claim that inclination must accord with commitment in order to achieve virtue. Hence, if the standard or purist interpretation turned out to be false about Aristotle, then the here defended notion of virtue would lose its tag “Aristotelian”. This would not, in the end, necessarily be an argument against the here defended notion of virtue, however. It would just turn out to not agree with Aristotle.^13^

Hence, let me motivate this Aristotelian threefold-distinction independently of whether it accords with Aristotle, between commitment, inclination, and action. It is helpful to distinguish between these three aspects involved in virtue, even if the distinctions don’t come from the history of philosophy. From above, we have seen that we can easily imagine—and probably know from experience—that all these constellations exist in daily life: People who tend to be self-controlled, those who tend to give in to their inclinations against their better judgment, those who aren’t necessarily committed to a good end in the first place, and those (rare ones) who seem to have their inclinations (mostly) aligned with their commitments to a good end. That is what we get from what I’ve called an Aristotelian framework of virtue. We might or might not have the intuition that only those who have their inclination aligned with their commitment are truly virtuous. That is, we might or might not want to say that the self-controlled is virtuous, too. This article sides with those who think true virtue means one’s inclination or habit is already aligned with one’s commitment, so self-control is not enough. This intuition comes from the idea that full virtue consists in a strong form of stability.^14^ Only if one acts well *stably*, is it full virtue. And this stability is only there—the idea goes—if one is not only committed to the virtuous goal, but has also formed one’s inclination in a way to be inclined to act in that way. That is what the distinction between self-control and virtue shows us. And it seems true that the most stable way to achieve virtuous agency is if one is not only committed to it, but if one has also habituated one’s inclination to act that way. To form one’s inclination, in Aristotle, consists in forming one’s feelings, emotions, and desires to react in the appropriate, fitting way in the individual case. Thus, in some sense, it is the forming of the perceptual and bodily capacities that creates inclination, or in short, an embodiment, an inscribing of the commitment into one’s body and affections. This is in contrast to a merely cognitive (and thus less stable) commitment. What would such an inscribing of the commitment into its body and affections be for a group? How could a group *embody* its commitments?

I suggest that this is exactly what we do when we institutionalize a group commitment. Institutionalization, as I understand it here, is the creation of policies, rules, and procedures that stabilize the group’s behavior, hence create an appropriate habit for the group.^15^ Creating policies, rules, and procedures exactly means to say for a group: ‘We want something like a body, an identity of the group, that endures even if the current individuals are exchanged or wouldn’t otherwise follow through on the commitment.’ That is what it means to transform a (mere) commitment into an *embodied* commitment for a group, and with it, a stable commitment. A group needs this embodied commitment (an inclination in the form of policies) in order to have something to compare its current actions to, to enable its stable good action. That is, creating policies for a group means to create an inclination.^16^ Hence, institutionalization is required for full virtue for groups. This does not mean that all institutionalized groups are virtuous. Any kind of commitment (also non-virtue-related and vicious ones) can become institutionalized in this way. But all virtuous collectives are institutionalized.

There could be two potential objections here: First, institutionalizing a commitment doesn’t *guarantee* its execution either, and second, why couldn’t there be other sources for strong stability in a group besides institutionalization, let’s say, if all the members have very strong desires to pursue the actions? First, one needs to distinguish strong stability from guarantees. It is hard to think of anything that is guaranteed that does not amount to a metaphysical necessity. In Aristotle’s terms, in the sublunary domain (the domain of human actions) the most we can achieve is “for-the-most-part regularities” (Vogt 2017). And it is this for-the-most-part regularity that virtue is aiming at. The argument here is thus not that with institutionalization we achieve a guarantee that a group acts according to their commitment to a good end. It is rather that without institutionalization, the group would not even achieve a for-the-most-part regularity, which we need for virtue.

And here is where the second objection comes in—why wouldn’t other sources suffice for a for-the-most-part regularity in this sense? Think of a group of friends who manage to meet weekly over twenty years. Their individual commitment is so strong so that they achieve for-the-most-part regularity. There are several ways how one can read such a group. Perhaps they have stability, but not a commitment to a good end—they might just meet because it is pleasurable. Hence, in that case it doesn’t amount to virtue because this is simply not a case in the normative realm. The argument of this article is not that we cannot achieve stability in any of our collective endeavors in life without institutionalization, but only in those in which we aim to achieve a virtuous end. It’s plausible that to achieve *a good end* in a stable way over an extended amount of time is more challenging than to achieve just anything stably over time. The argument is that for the former, we need institutionalization in the case of groups, while for the latter perhaps not.

But let’s imagine we have a group of friends who are committed to a good end—cleaning the neighborhood’s streets from its trash once a week. They’ve been doing this for twenty years, very stably. First, notice that while it’s possible that the involved individuals are friends, this is a contingent feature of that collective. The collective is constituted by the commitment to clean the streets, and by the practical identities that it takes to pursue such a commitment. If a group of friends generate such a collective, it might help its stability that they’re friends, but their friendship is (so far) a contingent aspect of it. Now, we can see the story go on in either of two ways. One, we can imagine that over time, the individuals change in that group (while the commitment and practical identities remain the same for the group), and so not all of them will be friends anymore. If they want the group to keep up stably pursuing the good end at that point, they must create some policies in order to keep up the group’s actions; policies that do not depend on friendship. Or second, alternatively, we can imagine the group using friendship *as a tool* to keep up the stability of its actions. That is, we can imagine that this group would only recruit new members with which they’re already friends, or that they’d expect any new member to also become a friend over time. In such a case, friendship is effectively the way in which they institutionalize their commitment. They don’t write down policies, perhaps, but instead institutionalize the group’s behavior by making friendship a necessary part of the involved practical identities. To count as taking up a practical identity of that group, one needs to become a friend with the other individuals involved in the group. Hence, again, it is institutionalization that brings about the required stability for virtue in the group. Friendship, and other tools that help structure one’s desires, can be a way in which groups institutionalize themselves. And indeed, many political parties, employers, and so on use similar tools to achieve ‘team spirit’ in that sense—they organize team outings, retreats, and so on, to use friendship (or good colleague-ship) as a way of institutionalizing the involved behavior, to achieve the required stability. We will further below look at comparisons of institutionalized and non-institutionalized collectives which try to virtuously pay attention. Before we can do this, however, we need a workable notion of attention. And as our examples will focus on collectives with hermeneutic tasks (e.g., scientists), we need a framework of attention that allows us to understand hermeneutic work as a form of attention.

## Attention as a Way of Understanding Hermeneutic Tasks

Sebastian Watzl (2017) argues that attention is, fundamentally, an activity of foregrounding and backgrounding mental contents.^17^ That is, attention is the activity of structuring mental contents into a priority-structure of foreground and background. If I pay attention to the scene in front of me, I foreground the black letters on my screen, and I background the coffee cup next to them. In this way, I create a priority structure between the letters (as they appear to me) and the coffee cup (as it appears to me).^18^

The usual way of paying attention is often (implicitly) thought of in terms of *focused* attention. We say “pay attention to the icy road”, and mean to say that one should focus on—or foreground—the fact that the road is icy, or the ice on the road. That is, focused attention consists in looking out for and thus foregrounding a specific aspect in the scene in front of me. I pay focused attention when I have a specific aspect in mind (e.g., “ice”, “black letters”) while creating a priority-structure, and I use my (habitualized) skills of foregrounding in order to achieve this focus.

Now, we are also capable of paying attention in a different way than with the goal of creating a focus. That is, we can do the foregrounding and backgrounding in a way that does not consist in having a specific aspect in mind while creating a priority-structure. It consists in the contrary: in deliberately *suspending* our usual ways of foregrounding, by backgrounding that which by itself has come to the foreground. This creates mental space for contents that are usually in the background. For example, if I stand in my garden, I can suspend my usual habit of foregrounding the berries on the branches of my plants. This will open up my mental space for things to come to the foreground that are usually in the background. For example, perhaps I now notice (foreground) the leaves, which usually stay unnoticed. We can call this ‘open-minded attention’ (henceforth called OMA), as a contrast term to ‘focused attention’.^19^ OMA consists in *suspending* one’s usual habits of foregrounding, so that a different mental priority-structure can emerge. Focused attention, by contrast, consists in *using* one’s usual ways of foregrounding in order to create a focus.

In the literature on attention, it is not widespread to find distinctions like the one suggested above, between focused attention and OMA. The usual kind of attention that is studied is focused attention. There are some notable exceptions, however. Gopnik (2009) and Adrienne Prettyman (2014, 2023) both discuss ‘diffuse attention’ as a kind of attention that stands in contrast to focused attention. Gopnik (2009) suggests a lantern model as opposed to the spotlight model to understand that kind of attention (ibid., p. 154). The idea here is that attention can also be spread more widely than with a clear and narrow focus. Instead of focusing, let’s say, on some berries in my garden, I can widen (or diffuse) my attention over the whole landscape scenery in front of me. In both Gopnik (2009) and Prettyman (2014, 2023), diffuse attention mainly consists in a wider focus, that is, the domain to which one pays attention is wider.^20^ That is, it is not a *different way* of paying attention, it is only that the attention’s domain is widened (hence, it is more like the light beam of a lantern than the one of a spotlight). Prettyman (2014, p. 72), along those lines, explicitly states that she still takes diffuse attention to be a form of selective attention—that is, attention that aims at selecting certain objects to be focused on as opposed to others. Hence, even though Gopnik (2009) and Prettyman (2014, 2023) consider other forms of attention than the narrowly focused one, their notion of diffuse attention remains within the framework of what I have called focused attention. It is just focused attention with a wider focus. It is not a form of suspending one’s focus altogether in attending.

However, as Prettyman (2014, p. 70) mentions briefly but then doesn’t develop further, there are also non-selective forms of attention. And if we understand diffuse attention along those lines, the concept comes much closer to our notion of OMA. The idea of a non-selective form of attention is well illustrated by Silvia Caprioglio Panizza (2022), when she presents us with Simone Weil’s concept of attention:

Attention consists of suspending our thought, leaving it detached, empty, and ready to be penetrated by the object; it means holding in our minds, within reach of this thought, but on a lower level and not in contact with it, the diverse knowledge we have acquired which we are forced to make use of. (…) Above all our thought should be empty, waiting, not seeking anything, but ready to receive in its naked truth the object that is to penetrate it. (Weil 1951)

The idea here seems to be, similarly to ours, that there is a kind of attention that consists in attentively waiting for something to strike one’s mind, as opposed to already selecting (foregrounding) certain features according to habit. So far, Weil’s notion of diffuse attention shares its core idea with our notion of OMA. However, Weil does not understand this process as a suspending of one’s habits of foregrounding. She understands it as a form of detachment, it seems, and a holding back of our thoughts. It is the idea of holding back mental content (thoughts, “diverse knowledge we have acquired”) from cluttering, as it were, our pure or empty attentiveness. This is not the same as OMA in our sense. In OMA we suspend our *habit* of how we foreground, not necessarily specific mental contents of ours. We try to undo our automatic process of foregrounding, which *might* result in the backgrounding of certain otherwise dominant mental contents, but it might also result in just seeing the same mental content in a different gestalt. That is, our notion of OMA is not so much about suspending certain dominant mental contents, but rather, about suspending a certain habit of how we foreground. This is a contrast to Weil’s concept of diffuse attention. But it is a rather subtle difference, and Weil’s concept might as well serve the same purposes as our concept of OMA.

We also find a similar notion of OMA to ours in empirical literature on attention, for instance in Waggoner’s (2021) research on virtue cultivation, specifically where she studies the role of “attention broadening” in virtue cultivation. Her research supports strongly the idea brought forward in this article: That we need not only focused, but also a form of OMA to achieve virtue. She concludes her study:

While attention narrowing often proves successful in modifying our behavior, problematic ethical consequences loom. By narrowing our attention, our phronetic-related skills can be threatened, leading to morally inappropriate actions. (…) cultivating virtue requires a balancing of broadened and narrowed attention. I suggested that by using both goal pursuit and OMM, we might have a better chance in avoiding problems that plague either in isolation, and so lead to the cultivation of virtue. (Waggoner 2021, pp. 20–21)

OMM stands for “Open Monitoring Meditation” and is a mental process very similar to OMA. Waggoner describes it while referring to research on how we can indirectly modify our cognition:

(…) OMM [is] an underexplored route for cultivating virtue. Upton (2017) explains that OMM is a kind of meditation where ‘the focus of the practitioner is to stay in the monitoring state, remaining attentive to any experience that might arise, without selecting, judging, or focusing on any particular object’ (pp. 336–337). OMM has also been defined as when ‘the individual is open to perceive and observe any sensation or thought without focusing on a concept in the mind or a fixed item; therefore attention is flexible and unrestricted’ (Colzato et al., 2012, p. 1). But, OMM is not simply letting one’s mind wander—it uses executive or top-down capacities and, ironically, still involves directing one’s attention (…). (Waggoner 2021, pp. 15–17)

So, here we have a description of an open form of attention that is close to ours: It is a deliberate suspending of one’s usual ways of foregrounding. By undoing one’s usual focus, other things, usually in the background or periphery, can come to the foreground. This undoing is not a passive ‘letting things go through the mind’, but an active undoing, an active suspending of one’s usual focus.

While there is this similarity, it is important to note that OMA as proposed in this article is not a form of meditation. It is one of two basic ways of paying attention, besides focused attention. The above presented similarity serves to show that the phenomenon of OMA has also been described in empirical research, that is, in work on how we can indirectly modify our cognition. To summarize, open-minded and focused attention are quotidian ways in which we pay attention, and which serve different functions in our lives. In some situations, we need focused attention, and in some we need OMA.

A worry might arise here: In what sense are these still two forms of attention, as opposed to just being two different kinds of mental activity? Perhaps, one might think, focused attention is ‘attention proper’, and what I call OMA isn’t attention at all; it is, rather, the mental activity of undoing attention, or something of this sort. Here are the reasons that speak against this objection. OMA is, just like focused attention, a way of actively (and perhaps indirectly, as Waggoner puts it) modifying our cognition. More specifically, just like focused attention, OMA is a way of modifying our cognition by manipulating what we foreground and what we background. Hence, what I suggest is a framework in which ‘modifying our cognition by way of manipulating what we foreground and background’ makes a mental activity be attention. This is the shared feature that focused and OMA have. Again, in focused attention we do this by *applying* the habits of foregrounding that we’ve acquired, and in OMA we do this by *suspending* those same habits. What is suspended in OMA is not the activity of foregrounding and backgrounding itself (i.e., that which makes OMA a form of attention is not suspended), but the *habit of how* we fore- and background by default is suspended, so that a different way of fore- and backgrounding can take over. That is why it seems right to say that OMA is just as much a form of attention as focused attention is, not an undoing of attention.

Let us now see the role these two kinds of attention play in understanding hermeneutic tasks. Roughly, one can distinguish between two kinds of hermeneutic challenges: if one lacks words for a phenomenon one encounters (hermeneutic gap), or if one has words but they seem inappropriate for the phenomenon at hand (hermeneutic distortion). A hermeneutic gap exists, for example, if I experience postpartum depression, but I have never heard of that concept before, and so cannot put a word on the kind of experience I am going through. I cannot tell others (or myself) ‘what it is’. A hermeneutic distortion exists, for example, if I experience sexual harassment, I don’t understand what is happening to me, but someone gives me a (distorted) concept under which to conceive of it, e.g., by telling me ‘this is (merely) flirting’.^21^ I now have words for the experience, but they don’t seem to be fitting. In either case, the hermeneutic gap and the hermeneutic distortion, I am confronted with a hermeneutic challenge.

We can conceive of scientific activity as having the goal of filling hermeneutic gaps and correcting hermeneutic distortions. Hence, if we want to understand, for instance, what it means for a group of scientists to pay attention virtuously, we should ask what kind of attention they pay when they fill hermeneutic gaps and correct hermeneutic distortions.

I argue that the filling of hermeneutic gaps can be best understood as a process of paying OMA, while the correcting of hermeneutic distortions can be best understood as a process of paying focused attention. In short, the idea is this: When we are confronted with a hermeneutic gap (we are confronted with a phenomenon for which we don’t have words), what we need to do is to suspend our usual, habitual ways of foregrounding, so that all the features, also the ones that are usually in the background and periphery, can be noticed (OMA). This is what will allow us to fill the hermeneutic gap (to find an appropriate concept). Imagine being in the state of postpartum depression without having a concept for it. If you suspend your usual ways of foregrounding (e.g. ‘I am unnecessarily sad’, ‘there’s no reason to feel this way’), you open up mental space for otherwise unnoticed aspects (e.g. ‘I haven’t met anyone outside of the house for months’, ‘my body has gone through a lot of changes’) to come to the foreground. This will allow you to gather a fuller set of descriptions of the phenomenon you are confronted with, and will eventually enable you to give your experience the words needed to fill the gap.

When we are confronted with a hermeneutic distortion (we are offered words for an experience, but they seem inappropriate), what we need to do is to look out for *specific* aspects of the phenomenon, that is, make precise use of our habituated skills of foregrounding, so that we can dissect what needs to be corrected from the words given to us (focused attention). Imagine being sexually harassed and being given ‘just flirting’ as a concept for it. If you now make precise use of your skills of foregrounding (e.g., ‘this feels uncomfortable’, ‘the notion of flirting has positive connotations’), you will eventually find out about the ways in which the offered concept is ill-fitting, which will enable you to find more appropriate descriptions to correct the distortion. Hence, as roughly illustrated here, we fill hermeneutic gaps with OMA, and we correct hermeneutic distortions with focused attention.

Let us thus assume this framework of attention as a way of understanding hermeneutic tasks. But how would that be done *by a collective*? And how would a collective pay this kind of attention *virtuously*? Let us thus come back to our framework of virtue for groups, where institutionalization plays the key role. We are now in a position to give an account of how a collective can pay attention virtuously, illustrated by cases of hermeneutic attention.

## Collective Hermeneutic Work

Let us imagine a collective that has the task of doing hermeneutic work. Fricker (2007) suggests that there are some professions that take up the majority of hermeneutic work in our society, such as journalism, politics, academia, and law.^22^ I call them ‘hermeneutic professions’. These professions have “practices by which collective social meanings are generated.”^23^ Now, for our example of a collective doing hermeneutic work, let us choose one of these professions, namely academia. Let us imagine there is a research group specifically tasked with picking up on hermeneutic gaps and distortions, in public discourse, in specialized fields, and of affected individuals who seek help. They are tasked with correcting the distortions and filling the gaps.^24^ How do they proceed?

As we’re imagining them as a research group, we’re tempted to already assume a few things: that it’s their job to do hermeneutic work (not a hobby, a personal interest, etc.), and that they’re part of an institution, a university, so already institutionalized. But let us backtrack from this and not yet assume those things. Let us instead imagine that a few people come together and say to each other: ‘You know, it seems that many human conflicts, suffering, or confusions are due to either a lack of concepts for their experiences, or due to having distorted concepts. Wouldn’t it be a useful thing for us to build a group that is specifically tasked with correcting and filling such distortions and gaps?’ So a few individuals together form the commitment of doing this hermeneutic work.^25^ Their joint commitment is what generates the group.^26^ The joint commitment out of which the group was built (to do this hermeneutic work) defines its identity, as it were. This will also determine what kinds of practical identities are required to be a member of this group. For instance, if the group is committed to filling hermeneutic gaps, the practical identity of the members will include a commitment to be able to suspend one’s usual ways of foregrounding, so that unknown features of an experience can be noticed (a commitment to OMA).

Having constituted such a group, we can now describe the process they have to go through when they try to pay attention virtuously. Let us describe the collective process for both focused and OMA, once with and once without institutionalization of the group.

### Non-institutionalized Collective Focused Attention

Let us imagine the group above that has formed to do hermeneutic work for others. They are now confronted with a hermeneutic distortion. Let’s say Carmita Wood^27^ comes to them and says that she experiences something at her workplace that makes her deeply uncomfortable. She can’t really understand why, because people call that behavior ‘flirting’, which has positive connotations. But it makes her feel so uncomfortable so that it makes her want to quit her job. And let’s imagine the group is being confronted with several similar reports. So, clearly, they think, there is evidence that there’s a hermeneutic distortion in the concept of flirting in these situations.

They might proceed now by going to observe those situations, or by collecting more reports of what was going on. What do they do when they observe or read reports? They now pay focused attention, because they are scanning the observations or reports for specific clues of what is uncomfortable in these situations. They are actively using their habits of foregrounding in order to look out precisely for these aspects.

As a collective, this is likely done in a division of labor. One member might talk to the affected individuals and ask them questions. Another member might systematize the reports and look out for commonly mentioned features, and so on. Now, if this group is not institutionalized, it is up to the individual members at any time whether or not they follow through on the commitment of the group to pay attention virtuously. So, even if the group is composed of the right combination of practical identities necessary for being virtuous, and therefore of the right kinds of commitments, if those commitments are not backed up with rules and policies, then the execution of those commitments is not stabilized enough. That is, in that case the group lacks the stable habit necessary for virtue.

We said that for a non-institutionalized group, there is nothing like an inclination or habit of the group that is independent of the current individual members. That is the sad thing about non-institutionalized groups: the group might have good commitments, but in any situation where the individual members are supposed to apply their skills, there’s nothing that pushes them to do so. There is nothing that stabilizes that these individuals will actually perform the practical identities that make up the group. There are no procedures in place that make them follow through if they don’t feel like doing it at the moment. If there is no habit of the group to do it, in the form of procedures and policies, then the execution of the group’s commitment in a specific case is always up to luck, in the sense that it is up to whether enough individuals follow through with the group commitment or not. The stability required for virtue is lacking. So, we would not call this group virtuous, simply because its performance is not stable enough for virtue, due to a lack of inclination of the group independently of the members’ motivation.

### Institutionalized Collective Focused Attention

The story in the institutionalized group is similar as above, but importantly different. Let’s say the group has institutionalized their commitment to pay attention virtuously in the form of procedures and policies. They have the policy, for example, to follow up on any request for hermeneutic corrections within a week of receiving the request. There are individuals who are paid (or in some other way legally bound) to execute certain tasks within a certain amount of time. Now, even if the individuals involved might lack the motivation to do so at a certain moment, the commitment *of the group* is habitualized in the form of procedures, and thus stable. The institutionalized procedures are the embodiment of the commitment of the group. There will, for instance, also be procedures in place about who will be in charge of the task next, in case an individual fails to show up. That is, it is clear who else will take up the same practical identity within the group, if a specific individual is missing or failing. Hence, collective focused attention—probably in a division of labor as described above—is institutionalized in this group, and thus there is an inclination of the group independently of individual members’ motivation to follow through on it. Hence, this collective can virtuously pay focused attention to hermeneutic distortions, and thus correct them stably.

### Institutionalized Collective Open-Minded Attention

For the collective process of filling hermeneutic gaps, let me start with the institutionalized, hence the fully virtuous group, and then show how the non-institutionalized fails to be so. Let’s imagine a group of medical doctors tasked with updating the list of existing mental illnesses, thus with a hermeneutic task. Their task is not only to list whatever illness is already recognized, but rather, their task is to find out whether there are any mental illnesses that aren’t as yet recognized. For that, they will have to pay OMA. That is, they will have to do the collective equivalent of looking out for the unknown, of actively maintaining a state of being prepared to notice yet-unknown things, by keeping their usual habits of foregrounding at bay. Their activity is to foreground that which is usually unseen in the background or periphery.

What is the collective equivalent of this activity? For instance, it could mean for this group that they have to reach out to marginalized people’s perspectives (the periphery) on certain mental problems, letting them describe the experience from their perspective. This might be the collective equivalent of ‘actively suspending one’s usual ways of foregrounding’. For example, imagine a time in which postpartum depression is still quite an understudied phenomenon because women’s health issues have traditionally been neglected. A collective with this task would then have to reach out to women undergoing mental problems after giving birth, in order to learn about the experience at all, instead of only asking the usual (male) subjects about their experiences with depression. By listening to women (the periphery) describing their experiences, they might find the unexpected and yet-unknown features and versions of the illness. That is, their OMA has to allow for new versions and aspects of the phenomenon to become recognized, through ‘letting the periphery speak’, that is, through actively suspending their usual ways of looking at things.

If this is an institutionalized collective, then the members of the collective don’t need to tell themselves each time ‘now we need to actively overcome our own filters and seek out marginalized perspectives’. Instead, there are procedures in place so that this is done habitually. For example, it has become a standard procedure to always first reach out to marginalized people’s perspectives when updating the list of mental illnesses. The commitment to OMA, then, is embodied in the policies and procedures that guide this collective’s activities. So, OMA is habitualized for this collective.^28^ This ensures the kind of stability that is required for virtue.

### Non-institutionalized Collective Open-Minded Attention

Let’s imagine the same group without institutionalization. This group would perhaps work similarly to what Fricker describes below, and what I call the ‘luckily neutral debate club’:

Imagine a debating society, the members of which are thoroughly prejudiced individuals. But their prejudices are all opposed and equally balanced, so that they cancel each other out in debate and the debate overall displays not prejudice but rather neutrality. (...) Such a debating society, then, is itself non-prejudiced, even while all of its members, and their contributions to the group’s activities, are prejudiced. (Fricker 2010, p. 8)

Fricker uses this example to illustrate that a group can have a feature (neutrality) that none of its members has.^29^ What is interesting in this example is that we encounter neutrality of the group by a kind of invisible hand mechanism.^30^*It just so happens* that the prejudices of the members cancel each other out. This way of exhibiting neutrality wouldn’t be stable enough to count as virtue. There is nothing that stabilizes the cancelling out of the prejudices, and therefore the stable habit is missing. And that’s exactly the problem with non-institutionalized groups. Whenever we have a non-institutionalized group, we have to rely on an invisible hand mechanism for a desired feature to emerge (like neutrality or open-mindedness), which is up to luck. It is the same way with a non-institutionalized group that tries to pay OMA. Institutionalization is the process in which a stable habit replaces luck or an invisible hand mechanism.

In the case of the luckily neutral debate club, they would potentially be virtuous if the members don’t just happen to have prejudices that cancel each other out, but if there were rather a policy in place that members be always chosen in such a way that their prejudices cancel each other out (their practical identities would then, by design, be aligned in such a way that stabilizes neutrality). That policy would create a habit of the group to be neutral, as opposed to depending on luck. Transferred over to the case of OMA, in a non-institutionalized group we could imagine that it would sometimes so happen that the perspectives of marginalized people are being sought out. But then, this would happen by luck, hence sometimes it would, and sometimes it would not. That is, even if the group is committed to OMA, if there is no policy in place that stably guides the actions of the members, the group will not stably achieve OMA, because the virtuous habit is lacking. It will be a matter of luck whether or not the current members think of seeking out marginalized perspectives or not. Hence, an institutionalized policy needs to be in place, in order to stabilize the seeking out of marginalized perspectives. This policy, which institutionalizes (i.e., habitualizes) OMA, could also consist in only choosing members for the group who themselves belong to marginalized groups.^31^ Either way, what makes the open-mindedness of the group stable is the involved policy that ensures the taking in of marginalized perspectives—the collective equivalent of OMA, the mental process of foregrounding what is usually in the background or periphery.

### An Asymmetry Between Focused and Open-Minded Attention

The notion of stability that is required for virtue that emerges from the discussion above seems to be about systematicity. That is, we want some systematic good collective action for virtue, not just lucky (random) good collective action. I think this is required if collective action is also supposed to be able to address systemic issues, such as hermeneutic gaps and distortions, and not only isolated instances of them. So, the stability required in both open-minded and focused collective attention is about systematicity of how attention is paid, so that it is in a non-random way done well.

While this holds for both open-minded and focused collective attention, there seems to be an asymmetry between the two forms of attention about *in which aspect* the stability of doing it well can fail. To see this, let’s once again ask how a group could fail to pay focused and OMA. When a group fails to pay focused attention, it is because it fails to follow through on its commitment to use its foregrounding skills in a certain instance. That is, we said, in a non-institutionalized group it can happen that the members don’t follow through on the commitment of the group and fail to pay attention to the hermeneutic distortion. What they do, then, *is not to pay attention at all*. They don’t pay attention in a wrong way, but rather, they do not perform an act of attention at all. They don’t pay attention in a moment they’re supposed to.

When failing to pay OMA, by contrast, the group does pay attention, just not in the right way. That is, they may *fail to suspend their habits* of what to foreground, and thus not be able to pick up on the as-yet not recognized features. But nevertheless, they are performing an action (paying attention), just not open-mindedly (they might pay focused attention instead).

So, what we want to systematize or stabilize in collective focused attention is that the members *follow through* when an occasion occurs where this is needed. What we want to stabilize in collective OMA is that the members suspend their habits of foregrounding, thus *the way in which* they pay attention. How can this asymmetry between focused and open-minded attention be explained?

This asymmetry is not tied to it being a collective phenomenon, nor to our conception of virtue, nor to the fact that we’ve been using the specific example of hermeneutic attention. It is tied to an asymmetry between focused attention and OMA itself. Focused attention is, in a way, the more primitive or the natural way of paying attention. It might be thought that this is the kind of attention we naturally learn, from a certain age on, without instruction. We need to foreground some things and background others, and when we pay focused attention, we do this in a certain domain, without instruction. Open-minded attention, by contrast, is rather an educational or cultural achievement. To suspend one’s usual ways of foregrounding in order to pick up on the as-yet unknown features might not be something we’d naturally start doing without instruction. The fact that we can do this, however, in individual and in collective processes, shows that it is part of our culturally acquired repertoire.^32^ We learned to do this in the course of history perhaps, and to instruct others to do this, in order to achieve certain things that we otherwise wouldn’t, such as picking up on mental illnesses that have not yet been recognized.

So, focused attention is something we cannot help doing every once in a while, and perhaps also what we default to if we don’t manage to pay OMA. OMA, by contrast, is something we learn to do by instruction, thus an educational and cultural achievement. Perhaps, in terms of development of the respective skills, the skills to pay OMA build on the skills one has (first) acquired to pay focused attention. That is why the only way we can fail to pay focused attention is to *not* pay attention at all—in the collective case, when the individuals don’t follow through on the group’s commitment. By contrast, when we fail to pay OMA, we fail *in the way* in which we pay attention—perhaps falling back on focused attention—while we might still pay attention. In the collective process, this is the case when we fail to foreground marginalized perspectives. And that is why virtue consists in stabilizing the following-through in the focused case, and in stabilizing the suspending of the usual foregrounding-habits in the open-minded case.^33^

The same asymmetry is also the reason why it is easier to first describe focused attention non-virtuously, just as a mental process that sometimes naturally happens, and then to ask what it would mean to do this virtuously (that is why I described its non-institutionalized form first, then the institutionalized one). The opposite is true of OMA—it is easier to first describe its virtuous (institutionalized) form—as it is an achievement already when we do it—and then to ask what it would mean to fail while trying to achieve it (likely falling back on focused attention).

In Sect. 2 I responded to the worry that perhaps not both forms of attention really are attention—that is, perhaps only focused attention is really attention, the worry goes, and the open-minded form is only an undoing of it. I responded that both are forms of (indirectly) manipulating our cognition, and more specifically, both are (opposite) ways of directing how we foreground and background—and that is why they are both forms of attention. It seems clear to me that this conceptual framework for attention also survives the just described asymmetry between focused and OMA here. Even if one of the forms of attention comes more easily to us than the other—and thus might be a more primitive one—what unites them as forms of attention is that they are both (opposite) ways of directing how we foreground and background. There seems to be no problem in thinking that there are more primitive and at the same time more demanding (and thus culturally achieved) forms of attention, all the while both are a form of attention.

## Conclusion

We now have an answer to how collectives can pay attention virtuously. The main take-away is that a collective needs to be institutionalized so to have the required fitting inclination to pay attention virtuously. Institutionalization is for a group what the training of affections and desires is for an individual—a process of embodying one’s commitments.

We have seen this illustrated by examples of two opposite kinds of attention, focused and open-minded. In focused attention, what needs to be institutionalized for virtuous collective attention is that the members of the group *follow through* in paying attention in a moment when it is required. In OMA, what needs to be institutionalized, instead, is *the way in which* the group pays attention—that it really does suspend its usual ways of foregrounding, which means, in the collective case, that it really does include the perspectives of marginalized groups.

We have focused on hermeneutic attention in our examples, that is, the kind of attention we need in order to master hermeneutic challenges and tasks. What has been said about virtuous collective attention in this context carries over to other collective forms of attention, however. This is so because any form of virtuous collective attention needs to have a stable inclination to pay the right kind of attention. That is, there is no reason to think that in other domains of (collective) attention, stability were to play a lesser role in achieving a virtuous form of it. And as long as stability plays a crucial role in achieving virtue, a stable inclination in the form of procedures will be necessary for collective virtuous attention.

Hermeneutic attention might be the kind of attention that, in many cases, is *necessarily* pursued by a collective, because it is about addressing systemic, that is, collectively shared issues. Cases like the research group or the group of medical experts considered in this article show that some hermeneutic tasks can only be tackled by an (organized) group, not by individuals alone. The here developed framework of virtuous collective attention might thus provide a helpful starting point to think about any kind of necessarily collective attention. At the same time, it gives us an illustration of how to think about scientific endeavors, as one instance of (often) necessarily collective attention.

## Footnotes

1 For the purposes of this article, I use the notions ‘group’ and ‘collective’ interchangeably, although technically they are to be distinguished (but in our context their distinction does not make a difference).

2 The literature on non-summative accounts of group agency is considerable. Some examples are: Tollefsen (2002a, 2002b), Gilbert (2004), Ritchie (2013, 2015, 2020), Lahroodi (2007), Rovane (1997), Schmid (2014), Mason (2021).

3 Some of these examples are taken from Fricker (2010).

4 Importantly, Fricker (2010) herself does not take her notion of group virtue to be specifically Aristotelian – she wants it to stay neutral between motive-based and skill-based virtue, for example, and so it could be based on Stoic-style virtue, too. However, as the two necessary requirements end up being commitment to a good end and reliable execution in her account, it seems that in the end it is mainly the Aristotelian notion of virtue she has in mind.

5 Fricker distinguishes two bases upon which such reliable execution can lie, namely either skill-based or motivation-based reliability. That is, either the motivation to achieve the end is so strong that the execution is reliable, or the collective skills of the group are so well-formed that the execution is reliable. (cf. Fricker 2010, pp. 241–244). These further distinctions in Fricker’s account are not relevant for our purposes, however.

6 One could question here why we would prefer a non-summative account of group agency in the first place. Why wouldn’t a summative account be enough? The short answer is that there is a sense in which summative forms of collective agency are not a collective phenomenon at all. The interesting aspect about collective agency is exactly the fact that a group can behave differently than what the majority or each individual involved would do. That is, if one wants to know how group agency works, one is usually interested in these *genuine* groups, where something more than just an addition of individual motives is going on. Beyond this, there simply are collective phenomena that can only be understood non-summatively. As we will encounter later in the article, borrowed from Fricker: Imagine a debate club where each of the members is thoroughly prejudiced. The prejudices of each of them, however, happen to cancel each other out. The group as a whole is thus unprejudiced. The feature of being unprejudiced is true about the group, while it is not true about any of its members. Hence, this feature is clearly a phenomenon that only exists on the collective level, that is, it is not a sum of the features of the involved individuals. Examples like this show that genuinely collective phenomena exist, such that they can only be understood non-summatively.

7 See, for instance, on p. 242: “Let us now add to this group motive the requisite reliability condition; and voilà, we have a collective virtue.”, but no story is told at all as to how one ‘adds the requisite reliability’. And again on p. 246: “If we add that the group as a whole reliably achieves the end of the motive/skill, then the group as a whole possesses the relevant virtue.”, but nothing is said in the remainder of the article on how that could come about (both citations in Fricker (2010)).

8 There is a question whether ‘there is’ an individual ‘behind’ all our practical identities at all, or whether what it is to be an individual is to be a distinct combination of various practical identities. The latter view is sometimes called the onion-model (because there is no core ‘at the end’ of an onion, once one has peeled off all its layers), and is often preferred by sociologists. The former is sometimes called the core-model (because a core individual identity is assumed ‘behind’ the layers of practical identities), and is often preferred (or simply assumed) by psychologists’ models of character.

9 This is so even though Fricker says she “borrows” the term from Korsgaard (Ficker 2010, p. 238).

10 Note that practical identities thus have different, complementary roles for collectives and for individuals: For individuals, adopting practical identities is a way in which they can become part of a group or perform a role. For collectives, practical identities are an instrument to define how individuals can be part of them. That is why an individual can have practical identities that conflict with each other (in order to be part of various groups), while it would not make sense for a collective to create or define its component-practical-identities in a way so that they conflict with each other.

11 Aristotle does not discuss this, but one could conceptually distinguish between ‘self-controlled vice’ and ‘full vice’ – where in the former, one is inclined to eat healthy food, but one self-controlledly acts viciously (so, eats the sweets against one’s inclination), while in full vice one’s inclination accords with one’s vicious commitment (so, one is inclined to eat the sweets, and eats them). Also – to complete the picture – it is imaginable that there could be akrasia in relation to vice – that one is committed to the vicious goal (to eat sweets), that one is inclined otherwise (to eat healthy food), and then one acts according to one’s inclination (eats the healthy food).

12 For instance, see Callard (2017), McDowell (1979, 1996, 1998), Broadie (1991, 2002, 2010), Coope (2012), Cooper (1998, 2010), Drefcinski (2000), Foot (2002), Hardie (1968), Irwin (1988), Kraut (1988), Lorenz (2006), Moss (2011), Woods (1986). However, one needs to note that I have taken the liberty of using the terms ‘commitment’, ‘inclination (habit)’, and ‘execution’ in line with Fricker (2010), but not necessarily in line with how Aristotle’s terms are usually translated. What I have called ‘commitment’ is what is usually translated as ‘insight’ or ‘understanding’ (logos, phronēsis), what I have called 'inclination’ is sometimes translated like that, and sometimes as habit (hēxis), and sometimes as the affections or passions (ta pathē), and what I have called ‘execution’ or ‘action’ is not usually referred to with a noun, but with a version of ‘how the agent acts’ in Aristotle scholarship.

13 See Hirji (2019) for the point that what is today standardly thought of as neo-Aristotelian virtue ethics does not align with Aristotle’s ethics, even in some of its main tenets, either. As Hirji (2019) points out herself, this does not mean that neo-Aristotelian virtue ethics is not convincing for independent reasons. See Hursthouse & Pettigrove (2023) for a formulation of standard neo-Aristotelian virtue ethics. And see Pettigrove (2018) for the point that we can think of virtue ethics also in completely non-Aristotelian terms – he highlights the four most important alternatives, namely an agent-based approach (Michael Slote), exemplarism (Linda Zagzebski), target-centered theory (Christine Swanton), and a neo-Platonic account (Robert Merrihew Adams).

14 Cf. *NE* II.4 1105a32-33, where Aristotle emphasizes stability as a criterion for virtue.

15 Note that to say that institutionalization creates an appropriate habit for the group does not commit us to say that institutionalization creates virtue. That is, we have to be careful here to remember that institutionalization – a stabilizing of the proper execution of a commitment – is only one among several necessary conditions for virtue. Institutionalization creates collective habits. When, and only when, the creation of these collective habits has been guided by a virtuous commitment, then institutionalization has co-created a virtuous collective habit, that is, a collective virtue.

16 This means, in some sense, that a group’s inclination is always in accordance with its initial commitments, because the inclination – in a collective – is created post hoc of (and according to) the commitment. This is a difference to individual virtue, where there is already an inclination before one has formed any commitments (because there is already a body, a perceptual apparatus with its habits and so on, before any commitment.).

17 To understand attention fundamentally as an activity foregrounding and backgrounding has historical precedent and is one of the leading research streams in current literature. Besides Watzl (2011a, 2011b, 2011c, 2014, 2017), Adrienne Prettyman (2014, 2023) is also building on this idea, and the idea exists at least since Husserl’s (1983, 1991) remarks on attention, and Gurwitsch’s (2009a, 2009b) more detailed development of it. Hence, my referring to Watzl (2017) here is supposed to be understood as just one clear and explicit example of an account of attention as foregrounding and backgrounding.

18 This is an instance of *perceptual* attention. The same principle can be applied to non-perceptual kinds of attention too.

19 One might think that once the leaves come to the foreground, I again pay focused attention, as I create a focus on the leaves. In the end, not much hangs on whether we only call the suspending-aspect itself open-minded, or whether the mental process as a whole is called open-minded, including the moment of the new foregrounding. For simplicity, I find it easier to call the mental process as a whole open-minded, even if it includes moments of focus.

20 Prettyman (2014) actually considers four different ways in which to distinguish diffuse from focused attention, only one of which is properly described by contrasting a wider from a narrower focus. Her other ways of conceptualizing the contrast (e.g., global vs. local) are nevertheless still within the framework of selective attention, as she herself also makes explicit.

21 The example is borrowed from Fricker (2007), although she doesn’t call it ‘distortion’.

22 cf. Fricker (2007, pp. 155–156).

23 cf. Fricker (2007, p. 152).

24 It’s important to note that Fricker does not think of what I call ‘hermeneutic professions’ necessarily along the lines of such a deliberate group that fills hermeneutic gaps. Quite on the contrary – these professions most often create collective social meanings not in a deliberate way, but as a side product, as it were. Moreover, these are not necessarily critical practices (as they are not necessarily deliberate or even self-conscious in the first place), so they often create harmful collective social meanings (e.g., sexist, classist, racist ones).

25 Here, we thus have a clear example of a group that deliberately forms itself with the aim of doing hermeneutic work, and that’s the basis upon which they will be able to do it virtuously. One might worry, here, that it seems quite strong if only groups that deliberately set out to do hermeneutic work can do it virtuously. It seems by contrast that there are quite a few collectives out there that do hermeneutic work well, without deliberately having set out to do so. The main difference to keep in mind is between doing something virtuously, and to do it well. Just because one does not do things virtuously, doesn’t mean one cannot do it well. In other words, there most likely are collectives out there that do hermeneutic work well, while not having formed to do so. However, for it to be virtuously done, it needs to be deliberate (and stable). This does not mean that all the members need to always have the deliberate goal in mind, or even one of them at any given point in time. The commitment is institutionalized in a virtuous group; hence, the intention is institutionalized too, and hence a group counts as doing hermeneutic work virtuously also when as a result of its (deliberately created) procedures and none of the members do it very self-consciously in the moment. However, a group that never formed that commitment and institutionalized it cannot do it virtuously. That doesn’t mean they can’t do it well – just for virtue, the forming of the commitment is lacking, and also the stability that is required.

26 So, the *genesis* of a group might sometimes be summative in this sense, namely that several individuals co-align their commitments in a way that generates a group with a joint commitment. That does not mean, however, that, once the group exists, it is a summative group. Once the group exists, it can have independent commitments (and practical identities) from the individuals that constitute it. The same holds for inclination or habit: In the genesis of a group, perhaps the (good) inclination of the group depends on aggregating the (good) inclinations of the involved individuals. Once the group is generated, however, the maintenance of the group inclination can be independent of any individual inclination. And it is also not the case that all groups are generated in this summative way. We could equally well imagine an already existing collective to say: ‘It seems important that we create another collective to do this kind of work for us. Let us create this other collective, define the practical identities and commitments of which it shall be constituted, and then find individuals to fill these roles.’.

27 Fricker (2007) refers to Carmita Wood’s case when discussing the hermeneutic gap that existed back when Carmita Wood experienced sexual harassment before there was a name for it.

28 One might think that there is a sort of contradiction in institutionalizing open-minded attention in this way: Doesn’t a fixed procedure make it rigid, as opposed to open-minded? I argue that this is exactly not the case. Because open-minded attention is exactly not just *the absence* of focus, but an *active undoing* of focus, one needs to do something to pay OMA. What is being made stable with institutionalization is that one does those things that enable OMA. That does not make OMA itself in any way more rigid – it just ensures that it can happen at all. We need some form of procedural stability in order to reliably pay OMA as a group.

29 Importantly, she has also argued that this would not amount to group virtue – because obviously, the commitment to a good end is missing (Fricker 2010, p. 239).

30 This label is borrowed from Fricker.

31 This, in a less exclusive way, seems to be a motivation for affirmative action in many institutions.

32 Open-minded attention could be thought of as a cultural achievement analogously to how Heyes (2018) thinks of cognitive gadgets – like for example literacy. In Heyes’s account, cognitive gadgets, like literacy, are passed on to subsequent generations through social learning: “people with a new cognitive mechanism passed it on to others through social interaction” (p. 2). Open-minded attention could be a cognitive gadget in her sense – once some human beings have found out that it helps with certain tasks if we suspend our usual ways of foregrounding, they started teaching this to the next generation, too. Hence, open-minded attention then has become part of our culturally acquired cognitive repertoire, like literacy.

33 While there is this asymmetry in what makes one succeed in focused and open-minded attention respectively, it is true in both cases that ‘virtue consists in paying the right kind of attention’. On the one hand, one can fail by not paying attention in a situation in which one should (where ‘only’ focused attention would have been required). On the other, one can fail by paying the wrong kind (focused instead of open-minded) of attention. But both are ways of failing to ‘pay the right kind of attention’ in a certain situation. Contrast this with a different possible conception of virtuous attention, where the relevant criterion is not ‘paying the right kind of attention’, but rather, the criterion is whether one’s attention is ‘directed at a virtuous goal’ or not. That is, one could have a conception of ‘virtuous attention’ where there is nothing interesting to be said in *just how* one pays attention (focused, open-minded, wide-scope, narrow-scope, etc.), but good and bad forms of attention are only distinguished by whether they are in the service of a virtuous goal. This would mean that, for instance, as long as I’m committed to thoroughness in directing my attention, it doesn’t matter exactly how (open-minded, focused, etc.) I do it. In contrast, our conception of virtuous attention takes it that virtue lies partially in *how exactly one goes about* paying attention. But as mentioned in the introduction, also in our conception this alone is not sufficient for virtue. One other criterion is indeed a commitment to a virtuous goal – hence, for example, open-mindedly pursuing a vicious goal would not amount to virtue in our conception either.
